# The Three Subtypes of Tick-Borne Encephalitis Virus Induce Encephalitis in a Natural Host, the Bank Vole (*Myodes glareolus*)

**DOI:** 10.1371/journal.pone.0081214

**Published:** 2013-12-13

**Authors:** Elina Tonteri, Anja Kipar, Liina Voutilainen, Sirkka Vene, Antti Vaheri, Olli Vapalahti, Åke Lundkvist

**Affiliations:** 1 Department of Virology, Haartman Institute, University of Helsinki, Helsinki, Finland; 2 Department of Microbiology, Tumor and Cell Biology, Karolinska Institutet, Stockholm, Sweden; 3 Swedish Institute for Infectious Disease Control, Solna, Sweden; 4 Finnish Centre of Laboratory Animal Pathology, Faculty of Veterinary Medicine, University of Helsinki, Helsinki, Finland; 5 Veterinary Pathology, School of Veterinary Science and Department of Infection Biology, Institute of Infection and Global Health, University of Liverpool, Liverpool, United Kingdom; 6 Department of Veterinary Biosciences, Faculty of Veterinary Medicine, Helsinki, Finland; 7 Finnish Forest Research Institute, Vantaa, Finland; 8 Department of Virology and Immunology, Helsinki University Hospital Laboratory (HUSLAB), Helsinki, Finland; 9 Department of Medical Biochemistry and Microbiology, Uppsala University, Uppsala, Sweden; University of California, San Francisco, United States of America

## Abstract

Tick-borne encephalitis virus (TBEV) infects bank voles (*Myodes glareolus*) in nature, but the relevance of rodents for TBEV transmission and maintenance is unclear. We infected colonized bank voles subcutaneously to study and compare the infection kinetics, acute infection, and potential viral persistence of the three known TBEV subtypes: European (TBEV-Eur), Siberian (TBEV-Sib) and Far Eastern (TBEV-FE). All strains representing the three subtypes were infective and highly neurotropic. They induced (meningo)encephalitis in some of the animals, however most of the cases did not present with apparent clinical symptoms. TBEV-RNA was cleared significantly slower from the brain as compared to other organs studied. Supporting our earlier findings in natural rodent populations, TBEV-RNA could be detected in the brain for up to 168 days post infection, but we could not demonstrate infectivity by cell culture isolation. Throughout all time points post infection, RNA of the TBEV-FE was detected significantly more often than RNA of the other two strains in all organs studied. TBEV-FE also induced prolonged viremia, indicating distinctive kinetics in rodents in comparison to the other two subtypes. This study shows that bank voles can develop a neuroinvasive TBEV infection with persistence of viral RNA in brain, and mount an anti-TBEV IgG response. The findings also provide further evidence that bank voles can serve as sentinels for TBEV endemicity.

## Introduction

Tick-borne encephalitis virus (TBEV) is a zoonotic flavivirus that occurs on the Eurasian continent and causes tick-borne encephalitis (TBE) in humans [1]. TBEV is considered the medically most important arthropod vector transmitted virus (arbovirus) in Europe [2]. Three TBEV subtypes have been identified: European (TBEV-Eur), Siberian (TBEV-Sib) and Far Eastern (TBEV-FE) [3]. TBEV-Sib and TBEV-FE form two separate lineages, which share a common ancestor. These two subtypes have radiated considerably earlier than the TBEV-Eur strains that are circulating today [4], [5]. The course and severity of human disease appears to differ between the three subtypes. While for TBEV-FE, a mortality of 30% has been reported, TBEV-Eur and TBEV-Sib infections are fatal in only 1–2% [6] and 6–8% [7] of the cases respectively. However, TBEV-Sib has been associated with an elevated risk of recurrent infections in humans [7], [8]. Phylogenetic analysis allows identification of Baltic and Siberian sublineages of TBEV-Sib. These are potentially also discernable based on their pathogenicity to humans [5], [8].

TBEV is maintained in nature by *Ixodes* ticks; TBEV-Eur mainly in *I. ricinus* and TBEV-Sib and TBEV-FE in *I. persulcatus*. Ticks act as vectors and also constitute the main reservoir for TBEV [1], [9]. Small rodents, in Europe most importantly the bank vole (*Myodes glareolus*) and the yellow-necked mouse (*Apodemus flavicollis*) are considered as bridge hosts for TBEV between the different life stages of ticks, in a process called non-viremic transmission [2], [10]–[14]. Ticks can also acquire the virus when feeding on a viremic rodent host or via vertical transmission, although tick co-feeding on non-viremic or even immune rodent hosts is considered the most relevant route of infection for the TBEV ecology, at least for TBEV-Eur [2], [11]. Co-feeding is dependent on local microclimatic conditions and, consequently, the geographical distribution of TBEV is focal [1].

TBEV-RNA has been shown to persist in rodents in both TBEV-Eur and TBEV-Sib foci [13], [15]–[19]. Rodents have also been found to serve as excellent sentinels for TBEV foci [15], [20]–[22]. Furthermore, vertical transmission of TBEV-Sib has been demonstrated in the northern red-backed vole (*Myodes rutilus*) [23]. Still, the relevance of viral persistence in rodents for TBEV transmission and maintenance remains unclear.

We previously detected TBEV-RNA in organs of free-ranging *Myodes glareolus* and *Microtus agrestis* outside the tick-feeding season, during two subsequent winters, both in TBEV-Eur and TBEV-Sib foci in Finland [19]. In the present study, we have further assessed the kinetics of TBEV infection in a natural rodent host, the bank vole (*Myodes glareolus*). Experimental infection with all three subtypes was undertaken to allow characterization of the course of infection and persistence of TBEV including the comparison of the subtypes in a controlled approach.

## Materials and Methods

### Viruses

The following strains of the three known TBEV subtypes (TBEV-Eur, TBEV-Sib and TBEV-FE) were used in the present study: TBEV-Sib, Kokkola-8 [24]; TBEV-Eur, Isosaari-5 and TBEV-FE, Buryatia-169 [25]. All three strains originate from ticks that were collected by flagging by us. The virus strains have first been isolated and then passaged once in suckling NMRI-mouse brains at the Haartman Institute, University of Helsinki, using the same protocols and facilities.

Suckling NMRI-mouse brains were homogenized in Dulbecco's PBS+0.2% bovine serum albumin and further diluted in Hank's Buffered Salt Solution (HBSS; Life Technologies). Virus titers were determined by rapid fluorescent focus inhibition test (RFFIT) as previously described [26]. The three subtypes were used at equal titers predetermined by RFFIT. A virus copy number optimal for infection was determined in a pilot experiment, in which bank voles were inoculated with a ten-fold series following the same protocol as in the final experiments.

### Experimental infection of bank voles (*Myodes glareolus*)

Eighty-two colonized, inbred, pathogen-free, young and sexually mature *M. glareolus* voles were included in the study. All animal handling was in compliance with the guidelines of The Swedish Institute for Communicable Disease Control, Solna, Sweden, and the experimental studies were approved by the authority for animal study ethics in Stockholm (#N419/10). All efforts were made to minimize suffering and animals were euthanized immediately if any symptoms were seen.

Two infection experiments were undertaken, a first examining the acute phase of infection and a second to assess persistent infection (Table 1).

**Table 1:** Timescales of the experimental infection studies.

Persistence study												
Days post infection		18		53		83		109		133		168
Time, weeks post infection	0	3	6	8	10	12	14	16	18	20	22	24
Number of animals in total	39	39	39	30	30	30	30	21	21	21	12	12
Animals euthanized/strain	0	0	0	3	0	0	0	3	0	3	0	4
Animals euthanized in total	0	0	0	9	0	0	0	9	0	9	0	12
Blood sampling		x		x		x		x		x		x
Urine and excrements were collected at every mentioned time point throughout the persistence study.												

Acute phase infection study					
Days post infection	0	4	8	14	25
Number of animals euthanized/strain	0	3	3	4	3
Number of animals euthanized in total	0	9	9	12	9

In both studies, voles received 100 µL virus solution, containing 100 fluorescent focus-forming units (ffu), as a subcutaneous injection in the neck. Infected animals were housed in isolated cages under BSL-3 conditions with water and food provided ad libitum. Two uninfected animals served as controls in each study.

The studies were undertaken on groups of 3–4 animals infected with one of the three virus subtypes. For acute phase infection, animals were euthanized at 4, 8, 14 and 25 days post-infection (dpi), respectively (Table 1). However, two TBEV-FE infected individuals housed together were euthanized at 12 dpi due to severe acute disease. For the persistence study, voles were euthanized at 53, 109, 133 and 168 dpi (Table 1), except for one TBEV-Eur infected animal scheduled for euthanasia at 133 dpi that died at 110 dpi.

From all animals, cardiac blood was collected during euthanasia. In addition, voles from the persistence study were bled from the retro-orbital sinus at 18, 53 and 84 dpi. The blood was collected in Microtainer® tubes (BD) and spun to gain the serum fraction that was subsequently stored at −80°C. Voles were necropsied immediately after death and brain, spleen, lung, kidneys and uterus (female animals from the persistence study) were collected. Brains were cut in half longitudinally. One half of the brain, each half of spleen, lung and uterus as well as one kidney were stored at −80°C for RNA extraction. The other half of the tissues as well as one kidney was fixed in 4% paraformaldehyde (PFA; pH 7.4) for histopathological examination.

During the persistence study, urine and fecal samples were collected from 31 dpi until the termination of the experiment whenever animals were handled for cage cleaning or blood sampling. Samples were stored at −80°C.

All work with active virus or potentially virus-containing tissues was performed in BSL-3 facilities.

### ELISA

To confirm TBEV infection and monitor the production of TBEV-specific IgG, the commercial IMMUNOZYM® FSME (TBE) IgG All Species kit (Progen Biotechnik GmbH) was used according to the manufacturer's instructions.

### RNA extraction and real time reverse transcriptase PCR for TBEV

RNA extraction was performed using the TriPure isolation reagent (Roche Diagnostics Corp.) according to the manufacturer's instructions. From all animals except for those examined at 168 dpi, tissue samples were initially mixed with 1 mL TriPure Isolation Reagent and homogenized using a Tissuelyzer (Qiagen). From voles euthanized at 168 dpi, tissue samples were homogenized in 500 µL Dulbecco's PBS+0.2% bovine serum albumin. Subsequently, 300 µL of the homogenate was added to 1 mL TriPure Isolation Reagent and the RNA extraction completed according to the manufacturer's instructions. The remainder of the homogenate was stored at −80°C.

Feces samples were homogenized in 500 µl 0.89% NaCl by adding a glass bead and vortexing. The homogenate was centrifuged at 4000× g for 30 min at 4°C, using an Eppendorf centrifuge 5417C (Eppendorf). RNA was extracted from 140 µL of the feces homogenate supernatant and from urine samples, using the QIAamp Viral RNA Mini Kit (Qiagen) according to the manufacturer's instructions.

The serum samples underwent one freeze-thaw cycle before final freezing. RNA was extracted using the QIAamp Viral RNA Mini Kit (Qiagen) according to the manufacturer's instructions.

Immediately after RNA extraction, real-time RT-PCR for TBEV was performed as previously described [27], but with 150 nmol/L forward primer, 500 nmol/L reverse primer, 400 nmol/L probe and 25 µl reaction volume. PCR thermal cycling was performed using the ABIPrism 7900HT Fast System (Life Technologies). RNA concentration was determined using a NanoDrop spectrophotometer (Thermo Scientific).

### Histopathological and immunohistological examination

Tissue specimens were fixed in PFA for 72–96 h, then trimmed and routinely paraffin wax embedded. Sections (3–5 µm) were prepared and stained with hematoxylin-eosin (HE) for histological evaluation or used for immunohistological staining.

Immunohistology for the demonstration of TBEV antigen was performed on the brain of all animals, using a rabbit polyclonal antibody generated against the Hochosterwitz TBEV isolate (kindly donated by Prof. Franz X. Heinz, University of Vienna, Austria [28], [29] and the horseradish peroxidase method (EnVision™; Dako). Formalin-fixed and paraffin wax-embedded Vero E6 cell pellets infected with each virus strain and harvested at 8 dpi served as positive controls for the immunohistological examination. The antibody recognized all three TBEV strains and resulted in a granular cytoplasmic as well as a peripheral reaction in infected cells. Consecutive sections incubated with an unrelated rabbit antibody against *Toxoplasma gondii* served as negative controls.

Selected brains of voles sacrificed at 8 and 12 dpi that exhibited an inflammatory infiltrate were also stained for leukocyte markers (CD3 for T cells, CD79a for B cells and lysozyme for macrophages and neutrophils), to characterize the infiltrating leukocyte population, and were stained for cleaved caspase-3 to demonstrate apoptotic cell death, following previously published protocols [30], [31].

### Cell culture virus isolation

Vero E6 cells were incubated with homogenized (brain) tissue specimens from voles euthanized at 168 dpi that had been tested positive by RT-PCR. Cells were passaged up to 9 times, every 4–7 days. At each passaging, a proportion of cells was fixed on a microscope slide with acetone for 7 min, followed by an immunofluorescence assay (IFA) to detect infected cells, incubating slides with anti-TBEV rabbit polyclonal antibodies (raised against TicoVac Junior vaccine, Baxter) and polyclonal swine anti-rabbit FITC conjugate (Dako) for 30 min at 37°C.

Real-time RT-PCR for TBEV was performed as described above, on RNA extracted from cell supernatants using the QIAamp Viral RNA Mini Kit (Qiagen) according to the manufacturer's instructions.

### Statistical analysis

The persistence of the three TBEV subtypes in different organs of infected bank voles was studied using GLMM (generalized linear mixed modeling) with binomial error distributions and a logit link function. In the full model, the three TBEV subtypes (TBEV-Eur, TBEV-Sib and TBEV-FE), time as dpi, tissue type (brain, lung, spleen, kidney, serum), and all their two- and three-way interactions were used as fixed factors. To account for repeated measurements from the same voles, a random intercept was allowed for each animal. The full model was reduced by removing fixed effects sequentially if their inclusion did not decrease AICc (sample number adjusted information criterion) by more than two units [32]. All models were fitted using the Laplace approximation method (lmer function of lme4 package [33] in the R software [34].

## Results

### Acute phase infection study

The first, acute phase study targeted TBEV-infected bank voles between 4 and 25 dpi (Table 1). Three strains originating from ticks and representing the three subtypes of TBEV were used. Each group of 13 individuals was inoculated subcutaneously with one of the strains using 100 ffu of virus. Infected animals were housed in isolated cages under BSL-3 conditions.

All voles inoculated with TBEV-Eur or TBEV-FE produced specific IgG antibodies, i.e. seroconverted to TBEV and/or were shown to be systemically TBEV infected based on the presence of viral RNA in their organs. However, only 8/13 bank voles inoculated with TBEV-Sib were found positive for TBEV by either of the two methods (Table S1).

**Figure 1:**
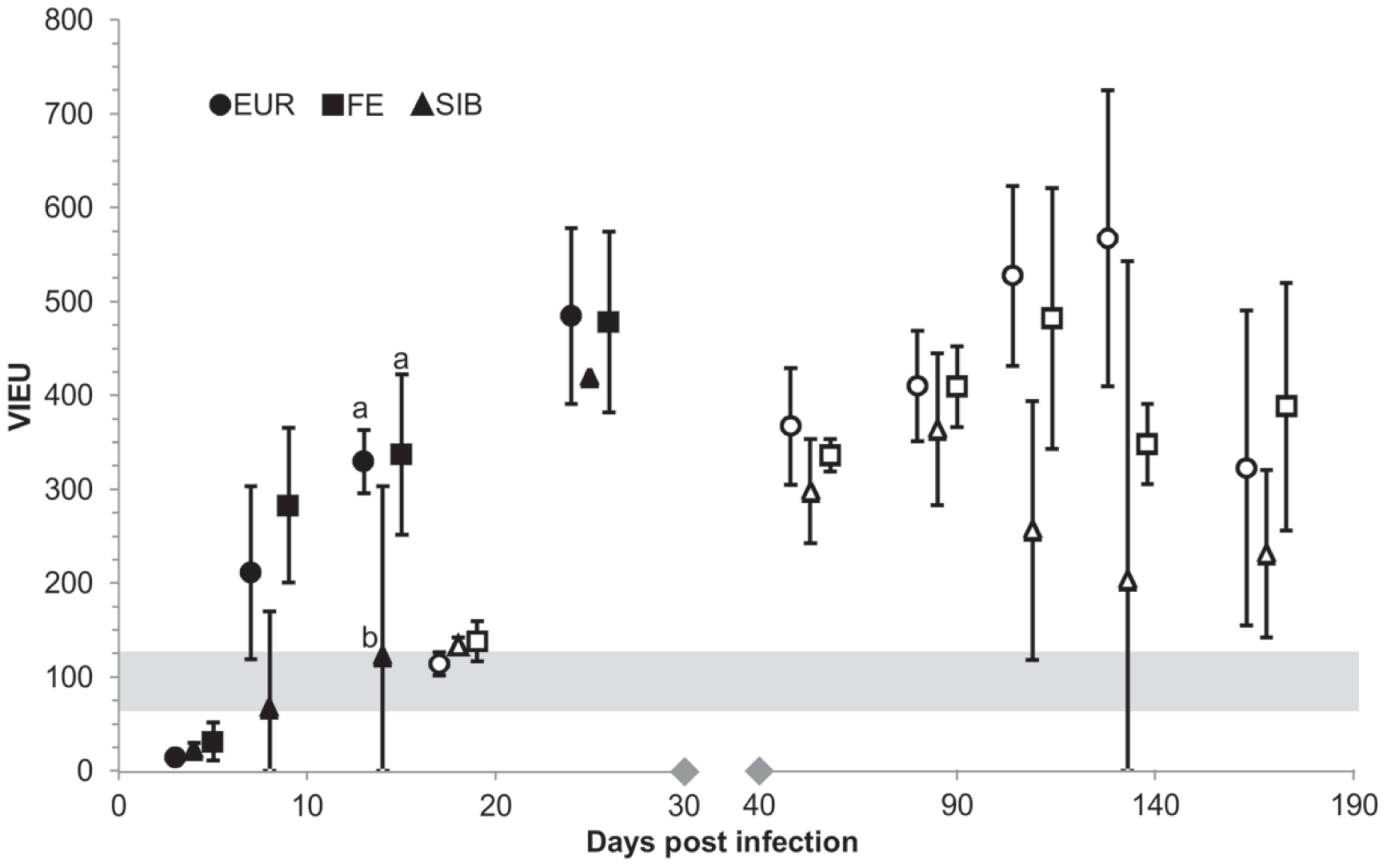
Antibody response in TBEV-infected bank voles. Antibody response (Vienna units of EIA, mean ±95% confidence interval) in TBEV-infected bank voles during the short-term (closed symbols) and the long-term experiment (open symbols). Strains that differ statistically significantly within a day post infection (P<0.05 in pairwise Tukey contrasts) are marked with a and b. The grey bar indicates the borderline area for EIA positivity. Notice the different scales on the separated parts of the x axis. EUR = European, FE = Far-Eastern, SIB = Siberian TBEV subtype.

**At 4 dpi**, all animals except for one TBEV-FE infected vole, were viremic, as shown by the presence of viral RNA in the serum. They all tested positive for viral RNA in the brain. Other organs were also positive for viral RNA, i.e.the spleen in all animals, the lungs in all but one TBEV-Sib infected animal, and the kidneys in all TBEV-FE, two TBEV-Eur and one TBEV-Sib infected vole. The histological assessment of the brain from TBEV-Eur and -FE infected animals did not detect any pathological changes, nor did immunohistology detect viral antigen expression. One TBEV-Sib infected bank vole exhibited a focal macrophage-dominated inflammatory infiltration in the frontal cortex, but viral antigen was not detected by immunohistology. The animals did not exhibit TBEV serum antibodies (Figure 1). However, spleens generally exhibited relatively large follicles with developing germinal centers that contained numerous apoptotic cells.

**At 8 dpi**, the EIA identified TBEV-IgG in all TBEV-Eur and -FE infected animals, but only in one of the three -Sib infected bank voles, and at borderline level (Figure 1, Table S1). However, in none of the animals was there histological evidence of secondary follicle formation in the spleen. All TBEV-FE infected animals were found to be viremic, based on the detection of TBEV RNA in the serum. In contrast, only one of the three TBEV-Eur infected animals and no TBEV-Sib infected animal exhibited viral RNA in the serum.

Apart from the brain, spleen, lung and kidney were found positive for viral RNA even if viremia was not detected, which would suggest infection of parenchymal cells in these organs. One of the TBEV-Sib infected individuals was negative in all tests (Table S1).

**Figure 2:**
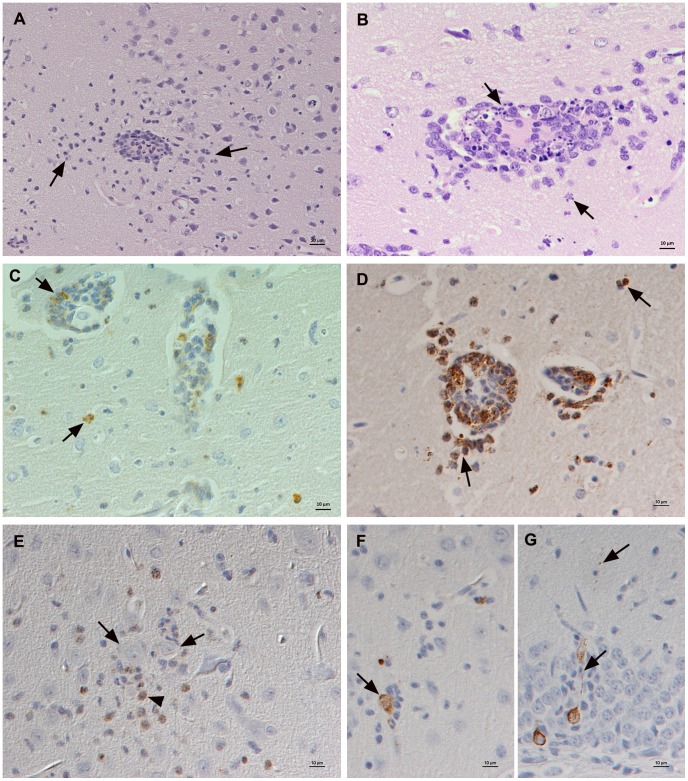
Brain of bank voles at 8 dpi. A–D. Cortex with moderate non-suppurative to mixed inflammation, mainly centred around veins. A. Vein packed with leukocytes that also emigrate from the vessel, form a perivascular cuff and are present in the adjacent parenchyma (arrows). HE stain; Bar = 20 µm. B. Vein with marked perivascular and parenchymal accumulation of leukocytes, numerous of which exhibit degenerative changes consistent with apoptosis (arrows). HE stain; Bar = 10 µm. C. Staining for cleaved caspase-3 confirms that leukocytes in both perivascular cuffs and adjacent parenchyma die via apoptosis (arrows). Peroxidase anti-peroxidase method, Papanicolaou's hematoxylin counterstain; Bar = 10 µm. D. Staining for lysozyme identifies the vast majority of infiltrating leukocytes in the perivascular infiltrates as macrophages and neutrophils. Peroxidase anti-peroxidase method, Papanicolaou's hematoxylin counterstain; Bar = 10 µm. E. Macrophages (lysozyme positive) are also found in parenchymal infiltrates and surrounding neurons (arrows) in satellitosis. Peroxidase anti-peroxidase method, Papanicolaou's hematoxylin counterstain; Bar = 10 µm. F, G. Expression of viral antigen. F. Cortex with scattered positive neurons, one of which is surrounded by microglia/macrophages (satellitosis). G. Hippocampus. Viral antigen in pyramidal cells and their processes (arrows). Horseradish peroxidase method, Papanicolaou's hematoxylin counterstain; Bars = 10 µm.

All TBEV-Eur and -FE infected voles, but only the IgG-positive -Sib infected vole exhibited viral RNA in the brain; these animals all showed a slight to moderate non-suppurative to focally mixed (meningo)encephalitis (Table S1) that was generally affecting the cerebral cortex, in most cases also the hippocampus and in one case the cerebellum. The inflammatory process was represented by the presence of leukocytes within smaller veins, and their rolling and attachment to activated endothelial cells, emigration and perivascular accumulation with spreading into the adjacent parenchyma (Figure 2A, B). In some cases, marked leukocyte apoptosis was observed (Figure 2B, C). Microglial nodules and diffuse microglial activation were also seen. Infiltrating leukocytes were mainly macrophages (lysozyme-positive), with a variable proportion of neutrophils (Figure 2B, D, E). In the TBEV-Eur and -FE infected voles, macrophages were also seen in the parenchyma, occasionally surrounding individual neurons (satellitosis; Fig. 2E), and there was occasional evidence of neuronal degeneration. Lymphocytes were less numerous among infiltrating leukocytes and were mainly T cells, whereas B cells were very rare and only seen in perivascular and leptomeningeal infiltrates. Viral antigen expression was observed in scattered neurons in the cortex (mainly internal pyramidal cell layer; Figure 2F), the hippocampus (pyramidal cells; Figure 2G) and the dentate gyrus (granule cells).

The antibody levels were increasing from 8 dpi until the end point of the study (Figure 1).

**Figure 3:**
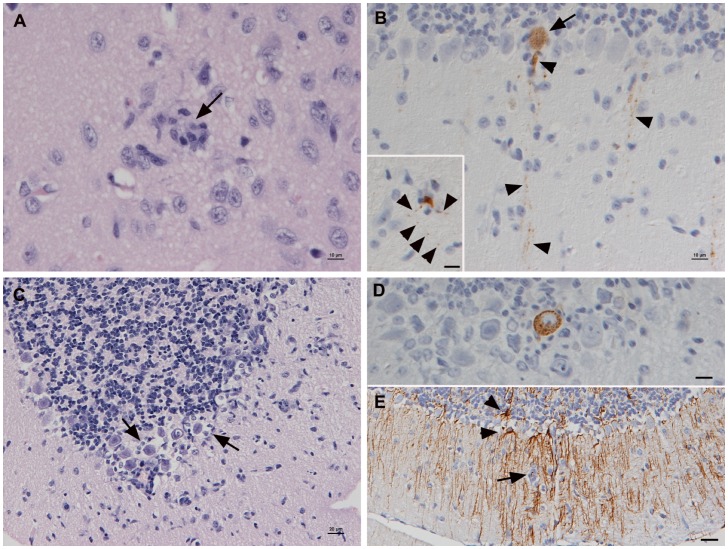
Brain of bank voles at 12 and 14 dpi. A, B. Animal euthanized at 12(arrow) and microglial activation. HE stain; Bar = 10 µm. B. Staining for TBEV antigen. Cerebellum with Purkinje cell expressing viral antigen in the cell body (arrows) and in cell processes (arrowheads). Inset: Viral antigen expression in cell body and processes (arrowheads) of an individual neuron surrounded by microglial cells/macrophages (satellitosis). Horseradish peroxidase method, Papanicolaou's hematoxylin counterstain; Bars = 10 µm. C–E. Cerebellum of animal scheduled sacrificed at 14 dpi. C. Satellitosis around Purkinje cells (arrows). HE stain; Bar = 10 µm. D. Purkinje cells expressing viral antigen, surrounded by microglial cells/macrophages (satellitosis). Horseradish peroxidase method, Papanicolaou's hematoxylin counterstain; Bar = 10 µm. E. Mild focal astrogliosis in area with increased cellularity (arrow: microglial nodule/infiltrating macrophages), indicated by the presence of GFAP-positive star-shaped reactive astrocytes (arrowheads) with numerous long processes. Peroxidase anti-peroxidase method, Papanicolaou's hematoxylin counterstain; Bar = 10 µm.

Two TBEV-FE infected voles that were housed together in one cage and scheduled for euthanasia at 14 dpi developed acute generalized symptoms and were euthanized at **12 dpi**. Both voles had developed antibodies against TBEV and exhibited viral RNA in brain, spleen, lung, and kidney, and one animal was also viremic (Table S1). The pathological findings in these voles were restricted to very mild non-suppurative encephalitis, mainly represented by scattered microglial nodules in cortex and cerebellum, with very rare satellitosis (Figure 3A). Viral antigen expression was more widespread and was observed not only in neurons in the cortex, but also in individual Purkinje cells in the cerebellum (Figure 3B), and in nerve fibres in the olfactory bulb in one animals. Staining for cleaved caspase-3 identified apoptotic cells in the microglial nodules, but no apoptotic neurons.

At **14 dpi**, all four TBEV-Eur, the remaining two -FE, but only one of the four -Sib infected animals were positive for TBEV-IgG antibodies in EIA (Figure 1). Both TBEV-FE, one -Eur- and one -Sib (IgG negative) infected vole were viremic at this stage (Table S1).

All non-viremic TBEV-Eur and -FE infected voles and one TBEV-Sib infected animal exhibited viral RNA in one or all of the tested organs, i.e. spleen, lung and/or kidney (Table S1). Also, regardless of the EIA results, when examined, the spleen of most animals exhibited well demarcated follicles with evidence of germinal center formation. Both TBEV-FE-infected voles harbored viral RNA in the brain, but without associated pathological changes; viral antigen was only found in one animal, in one weakly positive Purkinje cell. The brains of three animals inoculated with TBEV-Sib were negative for viral RNA and antigen and without any pathological changes, but the fourth animal exhibited a slight non-suppurative encephalitis and antigen expression together with positive PCR result for viral RNA (Table S1) in occasional neurons in cortex, dentate gyrus, and cerebellum, also in association with satellitosis. All TBEV-Eur infected voles exhibited viral RNA in the brain and two showed a mild non-suppurative encephalitis, affecting the cortex and hippocampus, and to a very low extent the cerebellum and brain stem. Viral antigen was detected in Purkinje cells in the cerebellum (Figure 3B, D) and, although generally only in low numbers, in cortex and hippocampus, mainly in association with satellitosis and/or glial nodules (Figure 3A–D) and with mild focal astrogliosis (Figure 3E). The third PCR-positive TBEV-Eur infected vole did not exhibit any pathological changes in the brain, but viral antigen expression was found in two neurons in the inner pyramidal layer of the cortex.

At **25 dpi**, all TBEV-Eur and -FE infected, and one of three TBEV-Sib infected animals, showed TBEV-specific antibodies in the EIA. Two of the three TBEV-Sib infected animals were negative in all tests. All IgG-positive voles exhibited viral RNA in brain and lung, some also in spleen and kidney, but viremia was only detected in the TBEV-FE infected animals (Figure 1, Table S1). The spleens of all animals, regardless of the EIA results, exhibited follicles with small germinal centers. Viral antigen expression was not detected in the brain of any vole despite often relatively high viral RNA titers. However, two TBEV-FE and two -Eur infected voles exhibited inflammatory changes in the brain, which in the -Eur infected animals represented a slight and mild non-suppurative (meningo)encephalitis respectively, with some perivascular lymphocyte cuffing in hippocampus and cerebellum as well as scattered small microglial nodules, whereas in the TBEV-FE infected animals, it was restricted to scattered microglial nodules.

### Persistence study

A separate study focused on animals that were sacrificed between 53 and 168 dpi (Table 1) and was expected to provide information on the persistence of the viruses in their rodent hosts outside the tick-feeding season. Groups of 13 bank voles were inoculated subcutaneously each with one strain.

All TBEV-Eur and -FE infected bank voles seroconverted to TBEV, whereas two of the 13 TBEV-Sib infected animals remained negative in the EIA. Both negative voles had apparently not become infected, as they were also negative for viral RNA in all tested organs (Figure 1, Table S1).

At **53 dpi** none of the animals exhibited viral antigen or any pathological changes in the brain, although TBEV-RNA could be detected by PCR. The other organs were also unaltered and only in two voles (one TBEV-Sib and one -Eur infected; both EIA-positive) did the spleen exhibit secondary follicles (Figure 1, Table S1).

From **109 dpi** onwards, TBEV-RNA was still detected in the brain (but only in the brain) of all animals (N = 9), regardless of the TBEV subtype used for infection (Table S1). Voles sacrificed at day 109 dpi did not show any pathological changes in the brain or any other organ, and there was no evidence of germinal centre formation in splenic follicles. Viral antigen was not detected in the brain.

**Figure 4:**
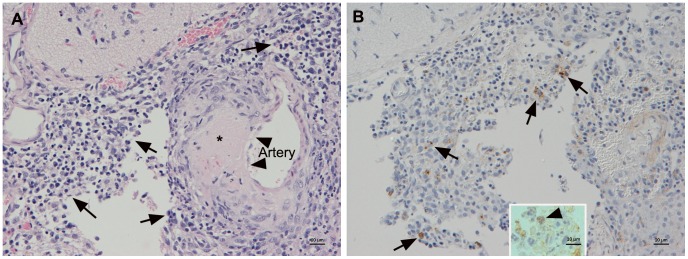
Brain of bank vole at 133 dpi. A. Marked focal, predominantly mononuclear leptmeningitis (arrows), surrounding a medium-sized artery that exhibits focal extensive fibrinoid necrosis of the wall (*) and focal degenerative changes of endothelial cells (arrowheads). HE stain; Bar = 20 µm. B. TBEV antigen is expressed cell free and by macrophages (arrows) and occasionally also by neutrophils (inset: arrowhead) in the infiltrate. Horseradish peroxidase method, Papanicolaou's hematoxylin counterstain; Bar = 20 µm (inset: 10 µm).

One TBEV-Eur infected animal scheduled to be euthanized at 133 dpi, was found dead at 110 dpi, without prior clinical symptoms. The brain was positive for TBEV RNA and the histological examination revealed a severe multifocal neutrophil-dominated and necrotizing ventriculitis and cortical leptomeningitis with the presence of viral antigen, both cell-free and within macrophages and occasional neutrophils (Figure 4). Adjacent to these infiltrates, a slight perivascular mixed cellular parenchymal infiltration was seen. A similar, although less intense inflammation was observed in another TBEV-EUR infected vole, which had been sacrificed as scheduled, at 133 dpi. In this animal, which also harbored viral RNA in the brain, the leptomeningeal infiltrates were found over the cortex and brain stem, surrounding a larger vessel that exhibited extensive fibrinoid necrosis (Figure 4A). Viral antigen was seen within macrophages and occasional neutrophils (Figure 4B). Also at **168** dpi, focal leptomeningeal lymphocyte accumulations were found at cortex and cerebellum in one of the TBEV-Eur infected animals (mild focal leptomeningitis; Table S1).

All other voles sacrificed at **133 dpi** and animals euthanized at 168 dpi did not show any pathological changes in the brain or any other organ. However, viral RNA was detected in the brain of two of the three TBEV-FE infected animals examined at 133 dpi and in one of the two remaining -Eur infected voles, whereas the -Sib infected voles were PCR-negative and no viral antigen was detected in the brain by immunohistology (Table S1). At **168 dpi**, the TBEV-Eur infected voles tested negative for viral RNA and only one of the four TBEV-Sib and –FE infected voles each tested positive. Also, RNA was detected in the serum of the TBEV-Sib infected vole still at 84 dpi (Table S1). Voles that were positive in both EIA and RT-PCR at 133 or 168 dpi exhibited higher IgG titers than those positive only in the EIA (Figure 1).

Vero E6 cell cultures inoculated with PCR-positive brain homogenates of animals sacrificed at 168 dpi tested negative for TBEV both in IFA and RT-PCR until 10 passages, confirming that cells were not infected.

The uteri were negative in the PCR at all time points and did not show any histological changes.

All non-infected control animals were negative in all tests and did not exhibit any histological changes in any examined tissues.

### Presence of TBEV RNA in tissues

**Figure 5:**
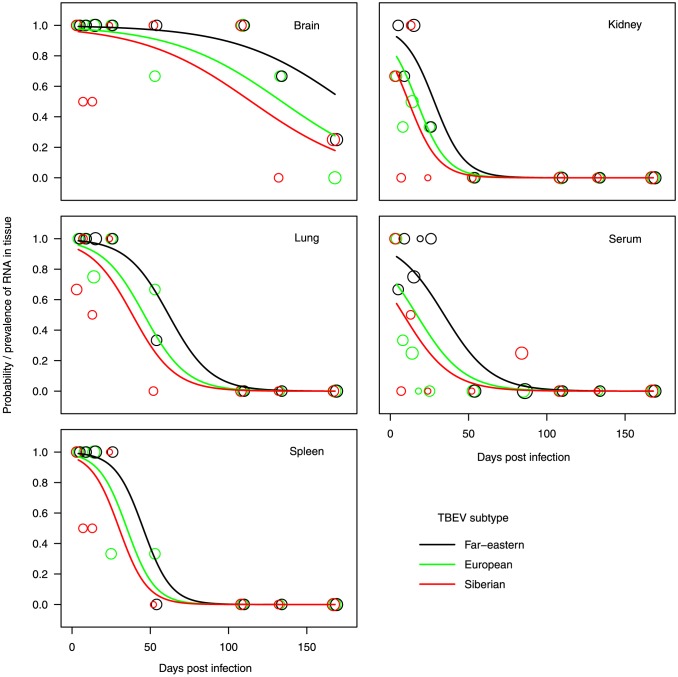
The observed proportion and the predicted probability of TBEV-RNA occurring in tissues of bank voles. Lines represent predicted values, i.e., estimates of fixed effects in Table 2. Circles indicate the observed proportions of TBEV RNA positive animals, their size being proportional to number of animals.

In the best supported GLMM (Table 2, Figure 5), fitted for the data from both infection trials, TBEV RNA was initially (4 dpi) found in the brains more often than in kidneys or serum. At this stage, lungs and spleens did not contain TBEV RNA any less frequently than the brain, but as the infection proceeded, the occurrence of viral RNA dropped significantly sooner in these organs than in the brain. Overall, TBEV RNA was significantly more often present in tissues of animals infected with TBEV-FE than in those infected with TBEV-Eur or -Sib. The latter two subtypes did not differ from each other in this respect (Table 2, Figure 5).

**Table 2:** The best supported GLMM analyzing the probability of a tissue sample of a TBEV-inoculated bank vole to contain TBEV RNA.

Explanatory variable	Factor level	Estimate (SE)^a^	z	P value
Intercept^b^		**4.86 (0.97)**	**5.0**	**<0.001**
Subtype	**European**	**−1.16 (0.43)**	**−2.7**	**0.007**
	**Siberian**	**−1.72 (0.49)**	**−3.5**	**<0.001**
Days post infection		**−0.03 (0.01)**	**−4.0**	**<0.001**
Tissue	Lung	−0.58 (1.19)	−0.5	0.627
	Spleen	−0.23 (1.26)	−0.2	0.854
	**Kidney**	**−2.34 (1.10)**	**−2.1**	**0.033**
	**Serum**	**−2.85 (1.02)**	**−2.8**	**0.005**
Days post infection:Tissue	**Lung**	**−0.05 (0.02)**	**−2.1**	**0.033**
	**Spleen**	**−0.08 (0.03)**	**−2.6**	**0.009**
	Kidney	−0.08 (0.14)	−1.9	0.052
	Serum	−0.04 (0.02)	−1.9	0.059

Variance attributable to a random effect of animal identity (71 groups) was <0.01 with standard deviation <0.01.

^a^ Estimates are given on logit scale and standard errors of estimates are in parentheses. Significant coefficients are in bold.

^b^ Intercept is calculated for brain tissue of a bank vole inoculated with Far-eastern TBEV subtype four days post infection.

## Discussion

TBEV has been shown to persist in wild rodents [13], [15], [16], [19], but the infection kinetics and the differences between the three subtypes of the virus have yet not been systematically studied. We infected bank voles (*M. glareolus*), which have been shown to carry TBEV in nature [19], [20] with all three TBEV subtypes to study prospectively and comparatively the infection parameters in an acute and prolonged setting under controlled conditions.

Animals were infected subcutaneously and, regardless of the subtype, the vast majority developed viremia, which was followed by infection of the brain 8 dpi at latest. Neurons were the only cells found to be infected, and the infection appeared to first occur in the cortex and hippocampus and then spread mainly to the cerebellum, where positive Purkinje cells were found on days 12 and 14 pi thus proving that TBEV is clearly neurotropic. Only very rarely was there evidence that the virus cause neuronal death in this natural host. At later stages, viral antigen was not further detected in neurons, although persistence of the TBEV RNA in the brain was confirmed by RT-PCR, supporting our earlier findings [19].

In all examined species so far, and both in natural and experimental infection, TBEV is exclusively neurotropic. In bank voles, we identified the virus in neurons in cortex, cerebellum and hippocampus/dentate gyrus. While viral antigen has been found in these locations in other species as well, the target neuronal populations are more varied in humans and dogs, and there appears to be a preference for the brain stem [28], [29]. However, subcutaneous infection of laboratory mice with TBEV yielded a similar neuronal infection pattern as in the bank voles [35].

The type and distribution of changes observed in the brain of infected bank voles in this study is very similar to that observed in other species after natural and experimental infection. We observed a macrophage and T cell mediated inflammatory response, but with neutrophil contribution. The latter was also observed in natural canine cases and in mice [29], [35], but was not a significant feature in human patients [36]. Early neutrophil recruitment is also observed in other flaviviral infections of the brain, such as West Nile virus (WNV) infection, where it has been attributed to marked elevation of neutrophil-recruiting chemokines by macrophages [37]. WNV replicates in neutrophils at high levels and is likely disseminated via neutrophils [37]. A similar mechanism appears possible for TBEV, since it has been shown to infect neutrophils [38], [39], although in the present study immunohistology did not detect virus in infiltrating neutrophils in the early stage of infection when there was evidence of cell recruitment into the brain parenchyma. Like in humans, viral antigen was mainly observed in intact and only rarely in dying, apoptotic neurons which further confirms that the virus is not likely to have a direct neuropathic effect and that direct activation of the apoptotic cascade is not a prominent mode of cell death in TBE [36]. This may be different in dogs and in laboratory mice, where substantial neuronal death has been observed, in the latter at least with a high inoculation dose [29], [35]. Interestingly, we observed a focal severe neutrophil-dominated leptomeningitis (and ventriculitis) in two animals late after infection (110 and 133 dpi, respectively) with the presence of viral antigen both cell-free and in macrophages, as well as evidence of immune complex vasculitis in one animal. It is possible that this is due to local confinement and replication of the virus, but cannot be readily explained.

Most animals in the present study did not develop any clinical symptoms and underwent scheduled euthanasia. This was most likely a consequence of the relatively limited inflammatory response that was generally seen. The results of our previous study on wild-caught rodents supports the finding that the infection is mainly asymptomatic also during natural infection of bank voles, as we could detect viral RNA in animals caught several months after the tick feeding season. We would not expect animals that had developed a significant disease or otherwise reduced fitness to stay alive until or over the winter [19]. So far, studies on the rodent reservoirs have not assessed whether TBEV infection (through the induction of encephalitis) can have an effect on the host rodent population via increased mortality. The burden of TBEV infection on host rodent population dynamics needs to be considered in a context of cost of parasite defences. Also, in the present study, animals were fed ad libitum, whereas in nature, limited nutrition may have an effect on immune functions [40]–[42]. A separate study addressing the effect of TBEV in a rodent population should be conducted in previously reported geographically restricted TBEV foci.

Rodents have been shown to serve as excellent sentinels for TBEV foci during tick-feeding season [15], [20]–[22]. In our earlier study, TBEV antibodies were seldom detected in wild rodents trapped in winter, 4–9 months after the probable infection period [19]. In the present study we could detect high levels of antibodies in animals infected with all subtypes until 168 dpi, together with morphological evidence of a systemic immune response to the virus, represented by secondary follicle formation in the spleen. These findings may indicate a difference between natural and experimental infection.

Chunikhin and Kurekov [12] reported viremia lasting 2–3 days in experimentally infected *M. glareolus*, while Knap et al. [21] suggested that the duration of viremia might have been underestimated in older studies. However, Chunikhin and Kurekov [12] described, that strains isolated from Buryatia, representing the TBEV-FE subtype, generally produced higher LD50 values on day 2–3 dpi than strains isolated in TBEV-Sib (Baltic) and TBEV-Eur regions. In our study, prolonged viremia was generally detected in TBEV-FE–infected animals and in one individual TBEV-Sib infected vole that was still TBEV-RNA-positive on 168 dpi. According to our present study, TBEV-FE-RNA was more often present in tissues independent of the time point. TBEV-FE is endemic in the area ranging from the Lake Baikal region to Northern Japan and the eastern part of Northern China. Some individual foci have been found also in the Baltics and Western Siberia [3], [43], [44]. The geographical distribution of *M. glareolus* covers only the endemic areas of TBEV-Eur and TBEV-Sib, while other Myodes species, *M. rutilus* and *M. rufocanus* inhabit the area endemic for TBEV-FE [45]. Nonetheless, because of the close relatedeness between *Myodes* species, the differences seen in TBEV-FE infection kinetics compared to the other two subtypes seen in our study were likely not due to different *Myodes* host species inoculated in this experimental infection.

We have not been able to infect suckling mice or Vero E6 cells with TBEV-RNA positive brain homogenates of persistently infected bank voles. This could be due to a possible loss of viral infectivity in association with its persistence in the brain. Similar results were obtained in rhesus monkeys after subcutaneous TBEV infection [46], [47]. So far, the determinants for persistence and its potential relevance for the transmission of TBEV remain unclear. Persisted TBEV could only be isolated from naturally infected *Myodes rutilus* after treatment with an immunosuppressant [48]. It could thus be speculated that a trigger, such as immunosuppression by hormones or stress, is needed to push the virus from persistence to viremia thereby becoming accessible for ticks. This hypothesis should be tested in natural host rodents. It is also possible that the route of infection (tick bite vs. injection) is of major relevance for the course of infection as tick saliva is known to contain several immunomodulating compounds [49] In order to investigate whether ticks can become infected directly by feeding on persistently infected voles, we undertook a pilot experiment as a part of the present study. We set colonized TBEV-free ticks (*Ixodes ricinus*) on voles that had been infected for 161–168 days. However, as most ticks failed to feed, the results remained inconclusive.

Rodents are often described as maintenance hosts for TBEV, but studies on TBEV-Eur support the hypothesis that ticks are both maintenance hosts and vectors for TBEV, while rodents serve only as bridges for non-viremic transmission between ticks during co-feeding [2], [9]. Predictive modeling of new TBEV-Eur foci in Europe is based on climatic conditions supporting *Ixodes ricinus* co-feeding [49]. However, this model cannot be directly applied to all three subtypes and different strains circulating in different biomes. Yellow-necked mice have been shown to support the non-viremic transmission between *I.ricinus* ticks better than bank voles [50]. Also, bank voles seem to develop resistance to feeding ticks thus hindering the successful feeding of ticks, which affects their potential to complete moulting. [51]. In Central European deciduous forests *Apodemus* mice are important hosts for TBEV. However, in Finland the dominant or even only rodent species trapped in known TBEV-Eur and –Sib foci have been *Myodes* and *Microtus* voles [19], [20]. Experimental infection experiments with *Apodemus* mice, similar to the current study, with all three subtypes are demanding, but would allow comparison between the two important rodent host species, *Myodes glareolus* and *Apodemus flavicollis*.

Besides non-viremic transmission and persistence in rodents, several other maintenance factors, for TBEV circulation and foci have been reported, for example transovarial transmission in ticks [52], sexual transmission in rodents [52], migratory birds [53], and deer density [54]. Studies on adaptation models of TBEV to different hosts and cell lines have also been published [55]–[57], and due to the complex transmission cycle of TBEV, the variation between individual TBEV strains, depending on the passaging history, time and site of isolation, and origin of the isolate should not be underestimated. In the current study, however, the strains were isolated from ticks in suckling mouse brain and passaged only once after that to minimize adaptation before inoculation of bank voles.

We hypothesized that there might be a difference in the transmission cycle and maintenance of the three TBEV subtypes and that persistence in bank voles could contribute to the TBEV maintenance in nature at least for TBEV-Sib and -FE. We also wanted to study the infection kinetics of TBE in a rodent hosting the virus in nature. The long duration of TBEV-FE viremia can be a potential maintenance factor, and may suggest a different transmission pattern as compared to TBEV-Eur. On the other hand, the relatively high proportion of a pure, likely innate phagocyte response (macrophages, neutrophils, and microglial cells) seen in the bank voles might allow a more rapid viral clearance in bank voles as compared to other species [28], [58]. Based on our results, it can also be concluded that the course of TBEV infection in a natural rodent host, i.e. the bank vole, does in principle not differ from the species which more often develop a fatal encephalitis, a feature we only observed in two infected bank voles. The extent and pattern of the neuronal viral infection and of the inflammatory reaction appears to be crucial for the outcome.

## Supporting Information

Table S1 Results by infected animal.
(PDF)
