# Supplementary material for: The Three Subtypes of Tick-Borne Encephalitis Virus Induce Encephalitis in a Natural Host, the Bank Vole (Myodes glareolus)
Source: PLoS One. 2013 Dec 13;8(12):e81214. doi: 10.1371/journal.pone.0081214 (PMC3862475; doi:10.1371/journal.pone.0081214)
Supplement: Table S1 — Results by infected animal. (PDF) [file pone.0081214.s001.pdf]

Table S1

Results by infected animal

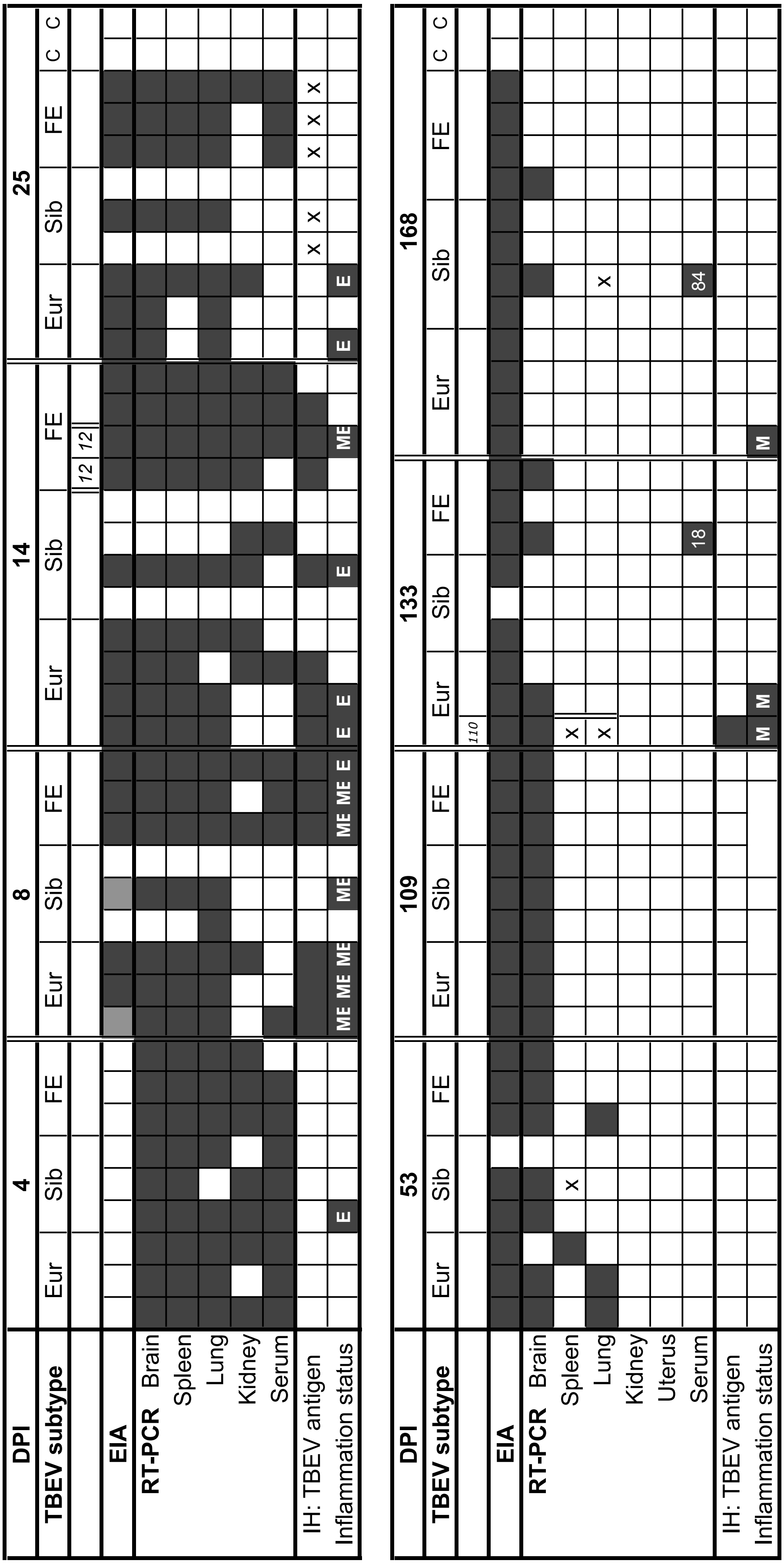

negative
borderline
positive

X No sample / result
IH Immunohistochemistry
ME Meningoencephalitis
M Meningitis
E Encephalitis
C Uninfected control

FE 14 dpi, 12:
Taken off on 12 dpi
Eur 133 dpi, 110:
Died on 110 dpi

Serum positivity dpi given, if sampling date other, than scheduled euthanazing dpi
